# The World Psychiatry Exchange Program in Iran: a unique academic and personal experience

**DOI:** 10.1192/bji.2023.11

**Published:** 2023-08

**Authors:** Cyrine Ben Said, Hela Ben Abid, Mohammadreza Shalbafan, Mariana Pinto da Costa

**Affiliations:** 1 Assistant Professor of Adult Psychiatry, Faculty of Medicine, Tunis El Manar University, Tunis, Tunisia. Email: cyrinebsaid@gmail.com; 2 Assistant Professor of Child Psychiatry, Faculty of Medicine, Monastir University, Monastir, Tunisia; 3 Assistant Professor of Psychiatry, Mental Health Research Center, Psychosocial Health Research Institute, Department of Psychiatry, School of Medicine, Iran University of Medical Sciences, Tehran, Iran; 4 Consultant Psychiatrist and Senior Lecturer, Institute of Psychiatry, Psychology & Neuroscience, King's College London, London, UK

**Keywords:** Education, training, transcultural psychiatry, exchange, low- and middle-income countries

## Abstract

The World Psychiatry Exchange Program in Iran is an academic experience we are delighted to share. As two participating early career psychiatrists, a local psychiatry faculty member manager, and the lead founder and international coordinator of the programme, we focus in this article on the unfolding of this new learning experience, the difficulties we encountered and the main lessons learned by the participants: commonalities and differences in training and practice in general adult psychiatry and child psychiatry in Tunisia and Iran, as well as in idioms of distress between the Arab and Persian cultures.

Psychiatry is arguably among the medical specialties more sensitive to the individuals’ cultural background and context. Reflections on the diversity of classifications, diagnosis and treatments highlight the importance of transcultural aspects of psychiatry.^1,2^ Although mental illness as a concept is universal, various studies have shown distinct aspects of mental disorders according to different cultures.^3,4^ These cultural variations have been shown to influence psychiatrists’ ability to detect, diagnose and appropriately treat mental health problems.^5^

As Western descriptions of the symptoms of mental disorders remain the most studied and widely shared, cultural specificities in other parts of the world, including in the Middle East and North Africa (MENA) region, may remain unclear for early career psychiatrists (ECPs) and trainees. Therefore, to better understand the transcultural aspects of mental disorders, opening up to different cultures is essential in the postgraduate training curriculum of psychiatry. Exchange programmes may offer ECPs the opportunity to live and work as locals for a few weeks, shadowing colleagues in overseas mental health services.

The World Psychiatric Association's World Psychiatry Exchange Program is open to members of the WPA Early Career Psychiatrists Section. In this article we share our reflections on participating in this unique academic experience in Iran, where it was hosted by the Department of Psychiatry at Iran University of Medical Sciences, Tehran. We write as two participating early career psychiatrists from Tunisia, a local psychiatry faculty member manager, and the lead founder and international coordinator of the programme.

## Programme of activities

The World Psychiatry Exchange Program in Iran entailed a 2-week programme of activities under the supervision of faculty members of the Department of Psychiatry at Iran University of Medical Sciences and involving the Iran Psychiatry Hospital, the Rasul-Akram Hospital, the Brain and Cognition Clinic and Tehran Psychiatry Institute.

Rasul-Akram is a general hospital, where ECPs could learn about the interactions between somatic and psychiatric diseases such as multiple sclerosis and psychosis or autoimmune diseases and depression. A day in a child psychiatry out-patient clinic was also scheduled in the Tehran Psychiatry Institute. Unlike with child psychiatry out-patients in Tunisia, psychotherapy is performed separately by specialised psychologists, whereas child psychiatrists are focused on psychoeducation and prescription. Medications such as methylphenidate were more prescribed and more available than is usual for patients in Tunisia.

The Iran Psychiatry Hospital is a spacious, well-organised psychiatric hospital, with five different educational groups: general adult, emergency, addiction, community-based and psychotherapy. The activities comprised visiting several wards, interacting with different academic psychiatrists and attending clinical psychiatric interviews translated into English. Exchange Program participants were also involved in daily medical meetings and discussion of scientific papers. The latter consisted of a meeting where a scientific article was presented by a trainee to their peers and to a senior chief, followed by a discussion about the possibility (or not) of applying the study findings in clinical practice in Iran. The psychotherapy ward of the Iran Psychiatry Hospital was a highlight, as there is no such department in the psychiatric hospital in Tunisia (Razi Hospital). Tunisian patients requiring psychotherapy are usually transferred to out-patient clinics near where they live, and (apart from the Department of Forensic Psychiatry) in-patient admissions are mainly for individuals with psychotic disorders.

In addition, 2 days were set aside to visit the Brain and Cognition Clinic and a primary care facility. The Brain and Cognition Clinic is a semi-state-funded facility with state-of-the-art technology for patient care and research in neurology, cognitive science and psychiatry. The participants experienced a peer-led model of research and were encouraged to collaborate on training, research and publications.^6^ Importantly, as participants, we were impressed by the way the World Health Organization's Mental Health Gap Action Programme (mhGap) was implemented in one of Tehran's primary care facilities, how systematic screening for mental disorders was performed for all patients and how data were computerised and collected.

## Challenges and opportunities

The language barrier is a challenge to consider during an exchange programme in psychiatry. Participating in an exchange programme in a country whose language we did not understand prevented us from attending long structured psychotherapy sessions, which cannot be simultaneously translated sentence by sentence. During an exchange programme one should also be mindful of the possibility of encountering logistical problems such as visa delays or issues with accommodation and public transport. Visiting the Iran University of Medical Sciences in Tehran and perhaps attending some of the courses there would also have been a great experience. Nevertheless, these 2 weeks were fulfilling and the lessons we learned during this exchange programme were invaluable.

## Adult and child psychiatry training curriculum and practice

The postgraduate training curricula for psychiatry trainees in Tunisia and Iran are different. In Iran, general adult psychiatry training lasts 4 years, which can be followed by 1 to 2 optional years in a subspecialty: child psychiatry, psychotherapy (usually focusing on psychodynamic therapies), psychosexual medicine, psychogeriatrics, liaison psychiatry, neuropsychiatry or addictions. In contrast, in Tunisia, child psychiatry and general adult psychiatry are two different medical specialties, each requiring 5 years’ training. During (or after) these 5 years, trainees can take 1- or 2-year courses – depending on the subject – in psychotherapy based on cognitive–behavioural therapies, brief therapies or interpersonal therapy, in sexology, addiction psychiatry, perinatal care, pharmacotherapy, forensic psychiatry and criminology, and adolescent psychiatry.

One of the core parts of the health system in Iran is the postgraduate medical training. There are daily training sessions in which one learns the techniques of a psychiatric interview, psychopharmacology and psychotherapy. Trainees’ supervision is provided not only by the seniors in the department where trainees are in rotation, but also by all seniors of the hospital, with daily meetings according to a pre-established schedule. Unlike the training of psychiatric trainees in Tunisia, where trainees have to do only a rotation in child psychiatry and neurology, in Iran there is also a mandatory rotation on a psychotherapy ward. In Iran, psychotherapy is integrated into psychiatric training programmes in most educational centres.^7^

Despite the good quality of medical education in both countries, young Iranian and Tunisian physicians are very interested in migration. Studies have reported that 69% of early career Tunisian family physicians and 83.7% of Iranian psychiatry trainees and early career psychiatrists had considered migrating. The reasons given are similar: work and financial conditions and the political context.^8,9^

## Idioms of distress

Observing how the expression of the same diseases can differ according to the culture (Persian and Arabic) was a unique learning experience.

There is some similarity between the Arabic language and Latin-originated medical terms, and we could therefore decipher parts of the psychiatric interviews before they were translated for us. The interviews seemed fluid and with rich and soothing non-verbal techniques, appropriate posture, calm gestures and a lot of empathy.

The content of the delusional statements made by patients with psychotic diseases had a different focus. Unlike Tunisian patients, there was less thematic content of bewitchment and ‘jinn’ possession. As these beliefs are widely present even in Tunisian patients with non-psychotic disorders, it is sometimes difficult to differentiate between cultural expressions of distress and psychotic diseases. Although believing in ‘jinn’ possession is not rare among the Iranian population, related delusions did not seem to be very common. Thematic content such as mysticism and jealousy seemed to be equally present in both cultures. Persecution may be more prevalent among Iranian patients with psychosis. This may be explained by historical facts, as Iranians have been persecuted by religious or political enemies for centuries.

Table 1 summarises some of the differences in services and idioms of distress in Iran and in Tunisia according to our experience.

**Table 1:** Differences based in our perception of services, training and idioms of distress in Iran and in Tunisia

	Iran	Tunisia
**Trainees’ education**		
Duration	4 years in general adult psychiatry plus 1–2 years in a subspecialty (as additional fellowship training after graduating from general adult psychiatry)	5 years in general adult psychiatry or 5 years in child psychiatry
Child psychiatry	A 2-year subspecialty after general psychiatry	A 5-year independent medical specialty
Psychotherapy	An 18-month subspecialty and 9 months during psychiatry training (mandatory)	2 years of complementary studies (during or after psychiatry training)
	Mainly based on psychodynamic therapies	Mainly based on cognitive–behavioural, brief or interpersonal therapies
Modalities	More interdepartmental collaboration	Each department has a specific training schedule
**Patients’ presentation in adult psychiatry**		
Content of delusions	More persecution	More possession and bewitchment
Personality characteristics	More identity problems, relationship instability and impulsiveness	More somatisation, victimisation, dramatisation
Substance use disorders	More methamphetamine use among psychiatric in-patients	More cannabis and alcohol use among psychiatric in-patients
**Interventions in child psychiatry**		
Medication	More availability and more prescription of methylphenidate and dextroamphetamines	Long-acting methylphenidate and dextroamphetamines are not available
Psychotherapy	Mainly practised by psychologists and other mental health professionals	Mainly practised by child psychiatrists
		More psychotherapeutic approach in treatment
**Primary care facilities**		
mhGap implementation	Well implemented and functional	Implementation in progress
Screening for mental disorders	Systematic	Not systematic

mhGAP, the World Health Organization's Mental Health Gap Action Programme.

## Conclusions

An exchange programme in a different context can be a strong pillar of psychiatric training for ECPs and trainees, enabling them to widen their scientific knowledge and to discover differences in idioms of distress between different cultures. It is of paramount importance to expand collaborations and to encourage more ECPs to gain inspiring academic and personal experiences.

## Data Availability

Data availability is not applicable to this article as no new data were created or analysed in this study.
